# Higher Protein Intakes Predict Leaner Body Composition in Weight-Loss Participants—Findings from the International Weight Control Registry

**DOI:** 10.21203/rs.3.rs-7915933/v1

**Published:** 2025-11-24

**Authors:** R Sayer, Tsz Kiu Chui, Lauren Fowler, Katie Ellison, Christopher Coleman, Satya Jonnalagadda, James Friedman, Susan Roberts, James Hill, Sai Krupa Das

**Affiliations:** University of Alabama at Birmingham; University of Alabama at Birmingham; University of Alabama at Birmingham; University of Alabama at Birmingham; Medifast Inc.; Medifast Inc.; University of Alabama at Birmingham; Dartmouth; University of Alabama at Birmingham; Jean Mayer USDA, Human Nutrition Research Center on Aging at Tufts University

## Abstract

**Background:**

A high protein diet combined with exercise is often recommended to promote fat loss and preserve muscle mass during weight loss. This secondary analysis evaluated the associations between dietary protein intake, physical activity, and body weight and composition among individuals engaged in purposeful weight loss attempts.

**Methods:**

This study included participants (n = 203) in a three-week ancillary study of the International Weight Control Registry, enrolled between August 2022 and January 2023. Average protein intake (g/kg body weight/d) was estimated from three 24-hour, multi-pass dietary recalls. Body weight, percent body fat (% BF), percent muscle mass (% muscle), and physical activity were monitored using study-provided consumer-grade devices. Multiple linear regression models were used to evaluate associations between protein intake and daily steps and their interaction with body mass index (BMI), % BF, and % muscle.

**Results:**

Total protein intake negatively predicted BMI (β [95% CI]=−0.51 [−0.62, −0.39], p < 0.001), % BF (β [95% CI]=−0.37 [−0.49, −0.26], p < 0.001), and positively predicted % muscle (β [95% CI] = 0.26 [0.20, 0.33], p < 0.001). Similarly, average daily steps negatively predicted BMI (β [95% CI]=−0.29 [−0.40, −0.17], p < 0.001), % BF (β [95% CI]=−0.23 [−0.35, −0.12], p < 0.001), and positively predicted % muscle (β [95% CI] = 0.14 [0.08, 0.21], p < 0.001). Total protein intake was significantly associated with BMI across all physical activity levels, with the strongest associations observed at 5,000 steps/day and weakening as the average daily steps increased.

**Conclusions:**

Findings broadly support the significance of high protein intake in achieving a lower body weight and a more favorable body composition (i.e., lower % BF and higher % muscle) for individuals previously engaged in weight loss. Additionally, people who are less physically active may require higher protein intake to maintain a lower body weight.

## Introduction

Reducing energy intake and increasing energy expenditure can result in clinically significant weight loss of 5–10%, which has been shown to improve cardiometabolic health outcomes.(1, 2) However, weight loss is often compromised by the loss of fat-free mass, including skeletal muscle, which accounts for approximately 25% of total weight loss.(3) A decline in skeletal muscle mass is associated with reduced quality of life and physical function, especially among older adults.(4) Therefore, maintaining skeletal muscle mass is crucial during weight loss.

Dietary protein plays an important role during weight loss by stimulating muscle protein synthesis (MPS), a metabolic process in which ingested amino acids are synthesized into muscle protein.(5) The current Recommended Daily Allowance (RDA), which is the recommended intake for healthy adults to meet basic nutritional requirements, is 0.8 grams of protein per kilogram of body weight per day (g/kg/d).(6) However, researchers have suggested increasing the recommended intake to at least 1.2 g/kg/d, especially for older adults to account for age-related muscle loss, as recent evidence supports the benefits of the proposed recommendation.(7) The current recommended intake for essential amino acids (EAA) is 184 mg/kg/d(8) while leucine is 42 mg/kg/d(6). Leucine is one of the nine EAAs, and the recommended intake of leucine makes up nearly 23% of the total EAA requirement, highlighting its importance compared to other EAAs. Evidence suggests that consuming 20–30 g of protein at a single eating occasion could maximize MPS.(9–11) Adequate intake of EAA, particularly leucine, also strongly stimulates MPS.(12, 13)

An energy-restricted, high protein diet combined with exercise is a frequently used strategy to promote fat loss while minimizing the loss of muscle mass during weight loss.(14–16) A high protein dietary pattern is usually defined as consuming more than the RDA, frequently specified in clinical trials as at least 1.0 g/kg/d of protein or more than 25% of daily energy intake from protein.(14) Data from the National Health and Nutrition Examination Survey (NHANES) showed that adults in the United States (US) typically consume approximately 16% of their total energy from protein, roughly 40 g per 1,000 calories, with this pattern remaining consistent across different age and sex groups.(17) Similarly, NHANES showed that non-Hispanic White individuals consumed 15.5% of total energy from protein, which was significantly lower than that of Hispanic (16.5%) and Asian (17.2%) individuals but did not differ from that of non-Hispanic Black (15.2%) individuals.(18) A meta-analysis shows that individuals consuming a high protein diet with energy restriction retain more fat-free mass, including skeletal muscle mass, and experience greater fat loss during weight loss compared to those on a lower protein diet across all adult age groups.(14, 16) Another systematic review and meta-analysis suggested that exercise provides additional benefits when combined with a high protein diet during weight loss than a high protein diet alone by better preserving fat-free mass, including muscle mass.(15) Although a substantial body of evidence reports protein intake in the general population and highlights the benefits of high protein diets and exercise during weight loss from clinical trials, more research is needed involving free-living individuals engaged in weight management to strengthen these findings.

This secondary analysis aimed to examine protein intake among individuals who have engaged in purposeful weight loss attempts and evaluate the associations between dietary protein intake, physical activity, and body weight and composition among these individuals. Protein intake was hypothesized to be similar across different age and sex groups. Since non-Hispanic individuals comprise most of the study population, protein intake was also hypothesized to be similar across racial/ethnic groups. Furthermore, higher protein intake (including EAA and leucine intake) and higher physical activity engagement would be associated with lower body weight and a better body composition profile (i.e., lower fat mass and higher muscle mass).

## Methods

### Participants

The ancillary study, used for this secondary analysis, included a subset of participants enrolled in the International Weight Control Registry (IWCR), a longitudinal study investigating factors related to successful long-term weight loss.(19) IWCR inclusion criteria include adults aged ≥ 18 years who have attempted or are planning to attempt weight loss. Detailed descriptions of IWCR recruitment and data collection have been published elsewhere.(19) Briefly, participants from the US were recruited through prior clinical trials, recruitment databases, healthcare networks, weight management centers, and community partnerships via email, printed flyers, and social media. Self-enrollment was also available through the study website (https://internationalweightcontrolregistry.org/). The IWCR protocol was approved by the Tufts University Institutional Review Board and is registered at ClinicalTrials.gov (NCT04907396). All participants provided electronic informed consent via the online consent form prior to enrollment. Data collection and management were conducted using REDCap hosted at the University of Alabama at Birmingham.(20, 21)

The ancillary study aimed to collect comprehensive and objective data on lifestyle behaviors as well as body weight and composition remotely. Eligible participants were existing IWCR enrollees residing in the US who completed baseline and 1-year follow-up surveys. Additional inclusion criteria were self-reported weight < 375 lb (170 kg) at the 1-year follow-up [to accommodate the 400 lb (181.4 kg) capacity of the Wi-Fi scale], ownership of a smartphone compatible with the Garmin Connect App, and access to 2.4 GHz Wi-Fi at home (necessary for the Wi-Fi scale). Enrollment for the ancillary study occurred between August 2022 and January 2023.

### Ancillary Study Timeline

**Figure 1:**
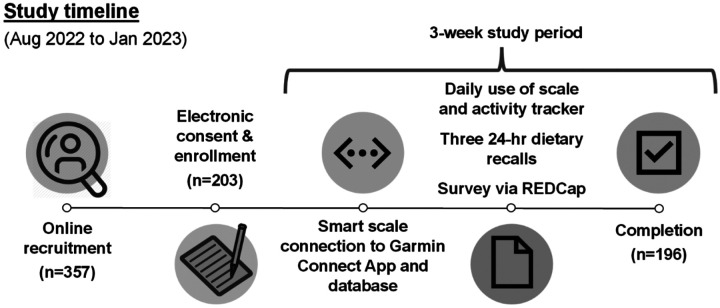
Ancillary Study Timeline.

The ancillary study timeline is shown in Fig. 1. Eligible IWCR participants were invited via recruitment emails with a link to the pre-screening form. Those meeting the ancillary study criteria were asked to provide informed consent and complete intake questionnaires via REDCap. Following enrollment, study staff mailed participants a Garmin Wi-Fi scale and activity tracker. Participants were instructed to connect both devices to the Garmin Connect App and the Validic data management platform, which enabled transmission of device data to the study team. Participants then completed three 24-hour dietary recalls. The three-week study period began upon completion of the first dietary recall. During the study period, participants completed the remaining two dietary recalls and questionnaires for diet and physical activity.

### Body Weight and Composition

Body weight and composition were measured using the Garmin Index^™^ S2 Smart Scale (Garmin Ltd., Olathe, Kansas, USA), which utilizes bioelectrical impedance analysis to assess body composition. The scale provided measurements for weight, body mass index (BMI), percent body fat (% BF), muscle mass (kg), and bone mass (kg). Percent muscle mass (% muscle) was calculated from scale measurements as [muscle mass (kg) / body weight (kg)] *100.

Participants were instructed to weigh themselves using the Garmin scale each morning for three weeks. Before weighing, they were asked to void, refrain from eating or drinking, remove shoes and socks, empty pockets, and remove clothing if possible. To maintain data integrity, participants were instructed not to allow others to use the scale during the study period. If either the scale or activity tracker remained inactive for 48 hours or more, participants received reminders via text or email to sync their devices with the Garmin Connect App. If inactivity persisted for more than 72 hours, research staff contacted participants by phone to troubleshoot technical issues.

### Dietary Intake

Each participant completed three multiple-pass 24-hour dietary recalls (two weekdays, one weekend) over the study period. Recalls were conducted by the Tufts Dietary Recall Team and analyzed using the Nutrition Data System for Research (NDSR). Dietary variables in this study were obtained from an average of the three diet recall days, including total protein intake (g/kg/d), EAA intake (mg/kg/day), leucine intake (mg/kg/day), and diet quality evaluated via the Healthy Eating Index 2015 (HEI) score. HEI scores range from 0 to 100, with higher scores reflecting better diet quality.(22)

### Physical Activity

Physical activity was tracked using the Garmin vívosmart^®^ 4 activity tracker (Garmin Ltd., Olathe, Kansas, USA). Participants were instructed to wear the device continuously, including during sleep, and only remove it for showering, bathing, or recharging. The data used for this study was the average daily steps.

### Statistical Analyses

All analyses were conducted with R version 4.5.0 (R Development Core Team 2025, Boston, MA, USA)(23) and the following packages were employed: tidyverse(24), doBy(25), DescTools(26), gtsummary(27), emmeans(28), psych(29), broom(30), ggplot2(31), and ggpubr(32). Significance thresholds were set at α = 0.10 for interaction tests and α = 0.05 for all other statistical tests.

The present study included data collected during the ancillary study period. For this analysis: 1) protein intake values (total protein, EAA, and leucine) represent the average across three 24-hour dietary recalls; 2) body weight, BMI, % BF, and % muscle were averaged over the ancillary study period; and 3) physical activity was evaluated as the average daily step count. Race and ethnicity were collapsed into a binary variable (Non-Hispanic White vs. Non-White), a three-category variable was created for age (23–44 years, 45–64 years, and 65 + years), and a binary variable was created for sex (male vs. female). Prior to analysis, data were examined for errors, patterns of missingness, and normality. Non-normally distributed variables were transformed as appropriate to meet model assumptions, and continuous variables were mean-centered and scaled prior to regression analysis.

Descriptive statistics were calculated as mean (SD) for continuous variables and n (%) for categorical variables for the overall sample. Pearson’s Chi-squared test was used to 1) compare group differences across age and racial/ethnic groups for HEI scores and protein intake, and 2) compare differences in the number of participants meeting requirements for recommended levels. For differences across sex groups, Wilcoxon rank-sum test was used to compare HEI scores and protein intake, and Pearson’s Chi-squared test or Fisher’s exact test (for counts less than five) was used to compare the number of participants meeting recommended intake requirements.

Multiple linear regression models were used to evaluate associations between protein intake and physical activity with outcomes in body weight and composition, adjusting for age and sex. Model diagnostics included residual scatterplots to confirm assumptions for homoscedasticity and linearity between continuous predictors and outcomes, while histograms and Q-Q plots were used to verify residual normality. No evidence of multicollinearity was observed in any model (variance inflation factor [VIF] < 5). To test whether physical activity modified associations between protein intake and outcomes, interaction terms (protein intake x physical activity) were included in the regression models. To aid interpretation of the interaction estimates, marginal slopes for protein intake associations were estimated at four physical activity levels (5000, 7500, 10,000, and 12,000 steps/day), representing “physically inactive”, “moderately active”, “physically active”, and “very active”, respectively.(33)

## Results

### Participants’ Characteristics

Table 1 shows characteristics of the overall sample. Participants (n = 203) had a mean age of 53.7 years (SD = 13.5) and a mean BMI of 31.7 kg/m^2^ (SD = 8.04), with 84% self-reporting as female and 76% as non-Hispanic White. Most participants (78%) held a college degree or higher and had a prior history of attempted weight loss (87%).

### Protein Intake and Diet Quality

Table 2 shows the distribution of protein intake and dietary quality across different age, sex, and racial/ethnic groups. Intakes for total protein, EAA, and leucine were consistent across age groups, with no significant differences observed. Specifically, the mean total protein intake ranged from 0.94 to 0.96 g/kg/d across all ages, EAA intake ranged from 356 to 382 mg/kg/d, and leucine intake ranged from 71.2 to 73.2 mg/kg/d. Participants aged 65 and older had the highest mean HEI score of 61.2 (SD = 14.5), compared to the 23–44 age group, which scored 51.7 (SD = 11.0), and the 45–64 age group at 57.3 (SD = 14.1), p = 0.002. Similarly, mean intakes of total protein (Non-Hispanic White: 0.96 g/kg/d vs. Other races: 0.92 g/kg/d), EAA (Non-Hispanic White: 362 mg/kg/d vs. Other Races: 354 mg/kg/d), leucine (Non-Hispanic White: 73.0 mg/kg/d vs. Other Races: 70.7 mg/kg/d), and HEI score (Non-Hispanic White: 57.6 vs. Other Races: 54.2) showed no significant differences across racial/ethnic groups. Regarding sex differences, males had significantly higher absolute intakes of total protein (93.6 g/d vs. 74.3 g/d, p = 0.002), EAAs (35.5 g/d vs. 28.4 g/d, p = 0.005), and leucine (7.18 g/d vs. 5.71 g/d, p = 0.004) compared to females. However, when protein intake was expressed relative to body weight, no differences were found between males and females in total protein (males: 0.97 g/kg/d vs. females: 0.94 g/kg/d), EAA (males: 366 mg/kg/d vs. females: 359 mg/kg/d), or leucine (males: 73.8 mg/kg/d vs. females: 72.2 mg/kg/d). There was also no significant difference in HEI scores between males (58.9) and females (56.4).

### Protein Intake Compared to Guidelines

Table 3 compares the distribution of participants who meet current recommended protein intakes across age, sex, and racial/ethnic groups. For total protein, over half of all participants meet the RDA of 0.8 g/kg/d, but fewer than 25% report consuming ≥ 1.2 g/kg/d. Similar patterns are seen across age, sex, and racial/ethnic groups. Conversely, most participants meet the requirements for EAA and leucine intake across these groups. Specifically, for leucine, nearly 90% of participants aged 45–64, over 92% of those aged 65 and older, and more than 97% of males meet the RDA of 42 mg/kg/d. For EAA, a statistically significant difference was observed between age groups in meeting the recommendation, with over 94% of individuals in the 45–64 age group and over 95% in the 65 + age group met the requirement for EAA.

### Effects of Protein Intake and Physical Activity on Body Composition

Table 4 shows the results of the regression analysis examining the associations between dietary protein intake and body weight and composition. Total protein intake negatively predicted BMI (β [95% CI]= −0.51 [−0.62, −0.39], p < 0.001), % BF (β [95% CI]= −0.37 [−0.49, −0.26], p < 0.001), and positively predicted % muscle (β [95% CI] = 0.26 [0.20, 0.33], p < 0.001). Similarly, average daily steps negatively predicted BMI (β [95% CI]= −0.29 [−0.40, −0.17], p < 0.001), % BF (β [95% CI]= −0.23 [−0.35, −0.12], p < 0.001), and positively predicted % muscle (β [95% CI] = 0.14 [0.08, 0.21], p < 0.001). Similar effects were observed for EAA and leucine intake (**Supplemental Table 1)**.

A significant interaction between total protein intake and average daily steps was observed solely for BMI, but not for % BF or % muscle. The predicted marginal slopes demonstrated that total protein intake was significantly associated with BMI across all levels of physical activity. The associations were strongest at 5,000 steps/day and weakened as the average daily steps increased (Table 5). Additionally, significant interactions were also found between leucine intake and average daily steps for BMI and % muscle, as well as between EAA intake and average daily steps for % muscle (**Supplemental Table 1**). The marginal slope effects showed that leucine intake was significantly associated with both BMI and % muscle across all levels of physical activity, and EAA intake was also significantly associated with % muscle across all levels of physical activity (**Supplemental Table 2**).

## Discussion

This study examined protein intake patterns among adults, most of whom had previously attempted weight loss, and the associations between dietary protein, physical activity, and body weight and body composition. The study found similar total protein (g/kg/d), EAA (mg/kg/d), and leucine (mg/kg/d) intake across all age, sex, and racial/ethnic groups, with no significant differences found between groups. Most participants met the recommended intake for protein, EAA, and leucine, but few met the higher 1.2 g/kg/d protein recommendation. Additionally, higher dietary protein intake and more daily steps were independently associated with lower BMI and % BF, and higher % muscle. Furthermore, higher total protein intake was associated with lower BMI at all levels of physical activity (i.e., from physically inactive to very active). The associations were strongest at 5,000 steps/day and weakened as the number of daily steps increased.

IWCR participants demonstrated consistent patterns of protein intake across all age, sex, and racial/ethnic groups, with average intakes above 0.92 g/kg/d for total protein. Participants aged 65 years and older consumed comparable amounts of protein as the younger groups. The results of this study are consistent with those from the NHANES data. While the NHANES data show that US adults of all ages and sexes consume approximately 16% of their total energy from protein,(17) IWCR participants consumed slightly more, at 18–19%. It is important to note that the total protein intake was only slightly above RDA guidelines. Additionally, just over half of the IWCR participants in each age, sex, and racial/ethnic group met the RDA of 0.8 g/kg/d, and fewer than a quarter met the new proposed recommendation of 1.2 g/kg/d. This suggests that many individuals in the IWCR did not consume adequate quantities of protein to meet the recommendations. This is particularly concerning given that the average age of IWCR participants was 53.7 years, and over 85% had a history of weight loss. Adequate dietary protein intake is crucial for stimulating MPS and preserving muscle mass during weight loss.(14–16) Physiologically, MPS is stimulated by protein intake, particularly a high proportion of leucine, to synthesize muscle fibers.(12, 13) Research has shown that consuming 20–30 g of protein every three hours, four times a day, can maximize MPS and thus optimize muscle mass.(9–11) Therefore, the insufficient protein intake observed among IWCR participants emphasizes the need to ensure sufficient protein consumption in this population.

Among IWCR participants, higher protein intake and more daily steps were independently associated with a more favorable body weight and composition profile (i.e., lower BMI and body fat as well as greater muscle mass). Findings from this study are consistent with extensive evidence from lifestyle intervention studies. For instance, a meta-analysis of 20 randomized controlled trials by Kim et al. found that older adults who consumed an energy-restricted, high protein diet with over 1.0 g/kg/d of protein had a greater reduction in body weight and fat mass, along with a smaller loss of lean mass including muscle mass, compared to those on consuming less than 1.0 g/kg/d of protein.(14) Similar results were also observed in younger adults. (16) Moreover, a recent meta-analysis of 31 studies found that walking at least 7,000 steps per day can lower risks of various diseases, including cardiovascular diseases, type 2 diabetes, and cancer.(34) The current study found that the negative association between protein intake and BMI persisted across all physical activity levels, from physically inactive to very active, with the strongest effect at 5,000 steps per day—considered a physically inactive level—although the benefits attenuated with higher daily steps. The findings from this study indicated that high protein intake may be particularly important for lowering body weight in sedentary individuals while its impact may be less significant for those with higher physical activity levels. Physical activity itself is known to stimulate MPS and promote muscle health.(35) However, beyond approximately 1.6 g/kg/d, additional protein intake does not provide additional benefits.(36) Overall, these interconnected findings emphasize the importance of both increased protein intake and regular physical activity for achieving lower body weight and better body composition in individuals engaged in weight management.

### Strengths and Limitations

Findings from this study bridge the gap between existing research, including NHANES data and clinical trials, by providing real-world data on protein intake from free-living individuals with prior experience in weight management. Additionally, the study includes comprehensive data from adults across the US. There are several limitations to consider. Although multiple-pass 24-hour dietary recalls, the current standard, were used to collect dietary data, they only represent intake during a shorter time window and do not provide a comprehensive perspective on dietary patterns over a prolonged period. Furthermore, individuals with obesity, who comprise a significant portion of this study sample, are known to underreport food intake.(37) Individuals included in this study were primarily non-Hispanic White females, with the majority having a college education or higher, which may limit the generalizability of the findings. The IWCR recruited and collected data exclusively online. The ancillary study also required participants to have access to Wi-Fi at home and a smartphone to connect to the Garmin devices. These constraints restricted recruitment to individuals with internet access and strong digital literacy skills. Additionally, due to remote data collection, the primary outcome—body composition—was measured at home using a commercial scale with bioelectrical impedance analysis. These scales, typically used in a home setting, are less accurate, and measurements can be influenced by factors such as hydration and the manufacturer’s proprietary algorithm. To ensure the data was reliable and valid, participants were given clear instructions on how and when to use the scale.

## Conclusion

This secondary analysis found that adults engaged in purposeful weight loss attempts had similar protein intake across all age, sex, and racial/ethnic groups and met the established recommendations of 1.0 g/kg/d for protein intake. However, fewer individuals achieved the proposed higher protein recommendation of 1.2 g/kg/d. Additionally, higher dietary protein consumption and daily step counts are associated with lower BMI and body fat, as well as greater muscle mass. The findings suggest that individuals in the current study have protein intakes comparable to those of the national sample of healthy adults, and their intake aligns with results from clinical trials. Most importantly, this study broadly supports the association between higher protein intake and lower body weight and fat, along with higher muscle mass, in individuals who engaged in weight management. Moreover, people who are less physically active may require a higher protein intake to maintain a lower body weight.

## Supplementary Material

Supplementary Files

This is a list of supplementary files associated with this preprint. Click to download.

- 2025.9.30v5ProteinIntakeandBodyCompositionSupplementalTables.pdf

## Figures and Tables

**Table 1:** Participants’ Characteristics.

CHARACTERISTICS	Overall Sample (n = 203)
Age, years	53.7 (13.5)
Sex	
Male	33 (16%)
Female	170 (84%)
Race and ethnicity	
Non-Hispanic White	155 (76%)
Non-Hispanic Black	26 (13%)
Hispanic or Latino	11 (5.4%)
Other	11 (5.4%)
Education	
High School/GED	9 (4.4%)
Some College/Associate's Degree	34 (17%)
College Degree	74 (36%)
Advanced Degree	85 (42%)
Household income	
<$25K	18 (11%)
$25K–$49K	37 (23%)
$50K–$79K	44 (28%)
>$130K	55 (35%)
US region	
Midwest	28 (14%)
Northeast	48 (24%)
South	96 (47%)
West	31 (15%)
^a^ Weight, kg	87.5 (23.9)
^a^ BMI, kg/m^2^	31.7 (8.04)
^b^ Percent body fat	37.7 (5.70)
^b^ Percent muscle mass	30.7 (3.30)
^c^ Average daily steps	7,086 (3,674)
Weight Loss Condition	
Maintained weight loss	68 (33%)
Regained lost weight	109 (54%)
No weight loss	14 (6.9%)
First attempt or considering weight loss	12 (5.9%)

Data are expressed as mean (SD) for continuous variables or *n* (%) for categorical variables.

a n = 202;

b n = 192;

c n = 195.

**Table 2:** Dietary quality and protein intake across age, sex, and racial/ethnic groups.

CHARACTERISTICS	AGE GROUP				RACIAL/ETHNIC GROUP			SEX GROUP		
	23–44 years (n = 55)	45–64 years (n = 89)	65 + years (n = 52)	p	Non-Hispanic White (n = 149)	Other Races (n = 47)	p	Male (n = 30)	Female (n = 166)	p
HEI Score	51.7 (11.0)	57.3 (14.1)	61.2 (14.5)	0.002*	57.6 (14.4)	54.2 (11.5)	*0.214*	58.9 (15.6)	56.4 (13.5)	*0.516*
Total Energy Intake, kcal	1,782 (488)	1,724 (567)	1,631 (503)	*0.271*	1,718 (559)	1,708 (424)	*0.827*	2,133 (724)	1,640 (449)	< 0.001*
**Total Protein Intake**										
% total energy intake	18.1 (4.91)	18.6 (6.19)	19.3 (5.78)	*0.404*	18.7 (5.93)	18.4 (5.12)	*0.946*	17.9 (4.63)	18.8 (5.91)	*0.404*
g/day	79.4 (28.6)	76.8 (23.7)	75.8 (26.3)	*0.719*	77.7 (26.3)	76.0 (24.1)	*0.721*	93.6 (33.1)	74.3 (23.1)	0.002*
g/kg BW/day	0.94 (0.47)	0.96 (0.37)	0.94 (0.37)	*0.609*	0.96 (0.38)	0.92 (0.45)	*0.251*	0.97 (0.38)	0.94 (0.40)	*0.757*
**EAA Intake**										
g/day	30.4 (11.2)	29.2 (9.69)	28.8 (10.8)	*0.64*	29.5 (10.6)	29.2 (9.97)	*0.855*	35.5 (13.5)	28.4 (9.38)	0.005*
mg/kg BW/day	359 (184)	363 (140)	356 (147)	*0.727*	362 (148)	354 (174)	*0.315*	366 (151)	359 (155)	*0.846*
mg/g total protein intake	382 (15.7)	378 (23.4)	378 (21.0)	*0.59*	378 (20.8)	382 (21.0)	*0.177*	376 (19.5)	380 (21.1)	*0.279*
**Leucine Intake**										
g/day	6.14 (2.30)	5.90 (1.92)	5.78 (2.10)	*0.635*	5.96 (2.11)	5.86 (1.97)	*0.736*	7.18 (2.68)	5.71 (1.87)	0.004*
mg/kg BW/day	72.4 (36.9)	73.2 (27.8)	71.2 (28.4)	*0.677*	73.0 (29.6)	70.7 (34.0)	*0.284*	73.8 (29.4)	72.2 (30.9)	*0.871*
mg/g total protein intake	77.1 (3.51)	76.4 (4.84)	76.0 (4.49)	*0.409*	76.5 (4.59)	76.6 (3.83)	*0.962*	76.2 (4.37)	76.6 (4.43)	*0.618*

Data are expressed as mean (SD).

**Table 3:** Comparisons across age and race for distributions of participants who met recommended protein intake requirements.

CHARACTERISTICS	AGE GROUP				RACIAL/ETHNIC GROUP			SEX GROUP		
	23–44 years (n = 55)	45–64 years (n = 89)	65 + years (n = 52)	p	Non-Hispanic White (n = 149)	Other Races (n = 47)	p	Male (n = 30)	Female (n = 166)	p
**Total Protein Intake**										
*RDA (0.8 g/kg BW/day)* ^a^										
Met requirement	30 (54.5)	53 (59.6)	29 (55.8)	*0.818*	87 (58.4)	25 (53.2)	*0.646*	16 (53)	96 (58)	*0.647*
Below requirement	25 (45.5)	36 (40.4)	23 (44.2)		62 (41.6)	22 (46.8)		14 (47)	70 (42)	
*New Recommendation (1.2 g/kg BW/day)* ^b^										
Met requirement	13 (23.6)	22 (24.7)	11 (21.1)	*0.89*	37 (24.8)	9 (19.1)	*0.546*	6 (20)	40 (24)	*0.626*
Below requirement	42 (76.4)	67 (75.3)	41 (78.8)		112 (75.2)	38 (80.9)		24 (80)	126 (76)	
**EAA Intake**										
*Recommended intake (184 mg/kg BW/day)* ^c^										
Met requirement	46 (83.6)	85 (95.5)	49 (94.2)	0.031*	139 (93.3)	41 (87.2)	*0.309*	29 (97)	151 (91)	*0.474*
Below requirement	9 (16.4)	4 (4.49)	3 (5.77)		10 (6.71)	6 (12.8)		1 (3.3)	15 (9.0)	
**Leucine Intake**										
*RDA (42 mg/kg BW/day)* ^a^										
Met requirement	45 (81.8)	80 (89.9)	48 (92.3)	*0.196*	134 (89.9)	39 (83.0)	*0.302*	29 (97)	144 (87)	*0.213*
Below requirement	10 (18.2)	9 (10.1)	4 (7.69)		15 (10.1)	8 (17.0)		1 (3.3)	22 (13)	

Data are expressed as *n* (%).

a Total protein and leucine recommendation based on RDA in the Dietary Reference Intake^6^;

b New total protein recommendation based on Traylor et al.^7^;

c EAA recommendation based on the World Health Organization guideline^8^.

Actual protein intake were shown in Table 2.

Group differences were compared using Pearson’s Chi-square test.

**Table 4:** Results of linear regression analysis examining the associations between total protein intake, physical activity, and body weight and composition (n = 195).

	Outcomes								
	BMI			% BF			% Muscle		
Predictor	Estimate	95% CI	*p*	Estimate	95% CI	*p*	Estimate	95% CI	*p*
Main Effects:									
Total protein intake, g/kg BW/day	−0.51	−0.62, −0.39	< *0.001*	−0.37	−0.49, −0.26	< *0.001*	0.26	0.20, 0.33	< *0.001*
Average daily steps	−0.29	−0.40, −0.17	< *0.001*	−0.23	−0.35, −0.12	< *0.001*	0.14	0.08, 0.21	< *0.001*
Interaction:									
Total protein intake x Average daily steps	0.092	−0.02, 0.2	*0.098*	0.02	−0.08, 0.12	*0.633*	−0.048	−0.11, 0.016	*0.14*

**Table 5:** Predicted marginal slopes for the association between total protein intake and BMI (outcome) at values of average daily step count. Estimates were derived from linear regression models with a significant interaction term for total protein x average daily step count on BMI.

Marginal Slope Effect	Average step count (PA category)	Estimate	95% CI	*p*
Total protein intake (g/kg BW/d) - BMI	5,000 steps/day (Physically inactive)	−0.55	−0.69, −0.42	< 0.001
	7,500 steps/day (Moderately active)	−0.48	−0.60, −0.37	< 0.001
	10,000 steps/day (Physically active)	−0.42	−0.57, −0.28	< 0.001
	12,000 steps/day (Very active)	−0.38	−0.56, −0.20	< 0.001

## Data Availability

Deidentified data from this study will be made available (as allowable according to institutional IRB standards) by reasonable request to the corresponding author. Analytic codes used to conduct the analyses and other research materials used to collect data for this study are available in the public archive at PubMed Central.
